# Adoptive T cell therapy for solid tumors: current landscape and future challenges

**DOI:** 10.3389/fimmu.2024.1352805

**Published:** 2024-03-14

**Authors:** Víctor Albarrán, María San Román, Javier Pozas, Jesús Chamorro, Diana Isabel Rosero, Patricia Guerrero, Juan Carlos Calvo, Carlos González, Coral García de Quevedo, Patricia Pérez de Aguado, Jaime Moreno, Alfonso Cortés, Ainara Soria

**Affiliations:** ^1^ Department of Medical Oncology, Ramon y Cajal University Hospital, Madrid, Spain; ^2^ Department of Medical Oncology, The Royal Marsden Hospital, London, United Kingdom

**Keywords:** immunotherapy, adoptive cell therapy, T cells, car-t, TCR-modified cells, TIL therapy, melanoma

## Abstract

Adoptive cell therapy (ACT) comprises different strategies to enhance the activity of T lymphocytes and other effector cells that orchestrate the antitumor immune response, including chimeric antigen receptor (CAR) T-cell therapy, T-cell receptor (TCR) gene-modified T cells, and therapy with tumor-infiltrating lymphocytes (TILs). The outstanding results of CAR-T cells in some hematologic malignancies have launched the investigation of ACT in patients with refractory solid malignancies. However, certain characteristics of solid tumors, such as their antigenic heterogeneity and immunosuppressive microenvironment, hamper the efficacy of antigen-targeted treatments. Other ACT modalities, such as TIL therapy, have emerged as promising new strategies. TIL therapy has shown safety and promising activity in certain immunogenic cancers, mainly advanced melanoma, with an exciting rationale for its combination with immune checkpoint inhibitors. However, the implementation of TIL therapy in clinical practice is hindered by several biological, logistic, and economic challenges. In this review, we aim to summarize the current knowledge, available clinical results, and potential areas of future research regarding the use of T cell therapy in patients with solid tumors

## 1 Introduction

### 1.1 T cells, antitumor response, and immune evasion

The antitumor activity of our immune system is a highly sophisticated process with several regulatory and negative *feedback* pathways. When malignant cells are identified and attacked by macrophages and natural killer (NK) cells -components of the innate immunity-, aberrant proteins derived from the cumulative occurrence of mutations are released and phagocytosed by dendritic and other antigen-presenting cells (APC) (1). In the peripheral lymph nodes, these tumor-associated antigens (TAA) are exposed by APC through major histocompatibility complex type I (MHC-I) molecules to the T cell receptor (TCR) of naïve CD8+ T cells, leading to their activation. For this ‘immune synapsis’ to be successful, other co-stimulating receptors on the T cell membrane (such as B7) should be activated (2). At the same time, the interaction between MHC type II (MHC-II) molecules and the TCR of CD4+ T helper lymphocytes leads to the activation of B cells and subsequent production of antitumor antibodies (3), and unleashes additional mechanisms that elicit CD8+ T cells function and differentiation, including dendritic cell licensing and cytokine production (4). Once CD8+ T cells are activated, they travel to the tumor site and recognize TAA presented by MHC-II molecules on the surface of malignant cells, unleashing the effector phase of adaptive immunity, and ultimately leading to tumor cell death (4). The quantity and phenotype of these tumor-infiltrating lymphocytes (TILs) have been widely associated with the biological behavior, prognosis, and response to anti-cancer therapies in virtually all subtypes of solid cancers (5–7). The success of the effector phase is compromised by the inhibition of T-cell response by immunosuppressive cells from the tumor microenvironment (TME), including myeloid-derived suppressor cells (MDSCs), tumor-associated macrophages (TAMs), and regulatory T lymphocytes (T-regs) (8). Tumor cells are able to modulate their function and differentiation through the activation of NF-κB and STAT3 signaling pathways, inducing the release of immunosuppressive cytokines (IL-6, IL-10, TGF-β) that inhibit TILs antitumor activity (9).

**Figure 1:**
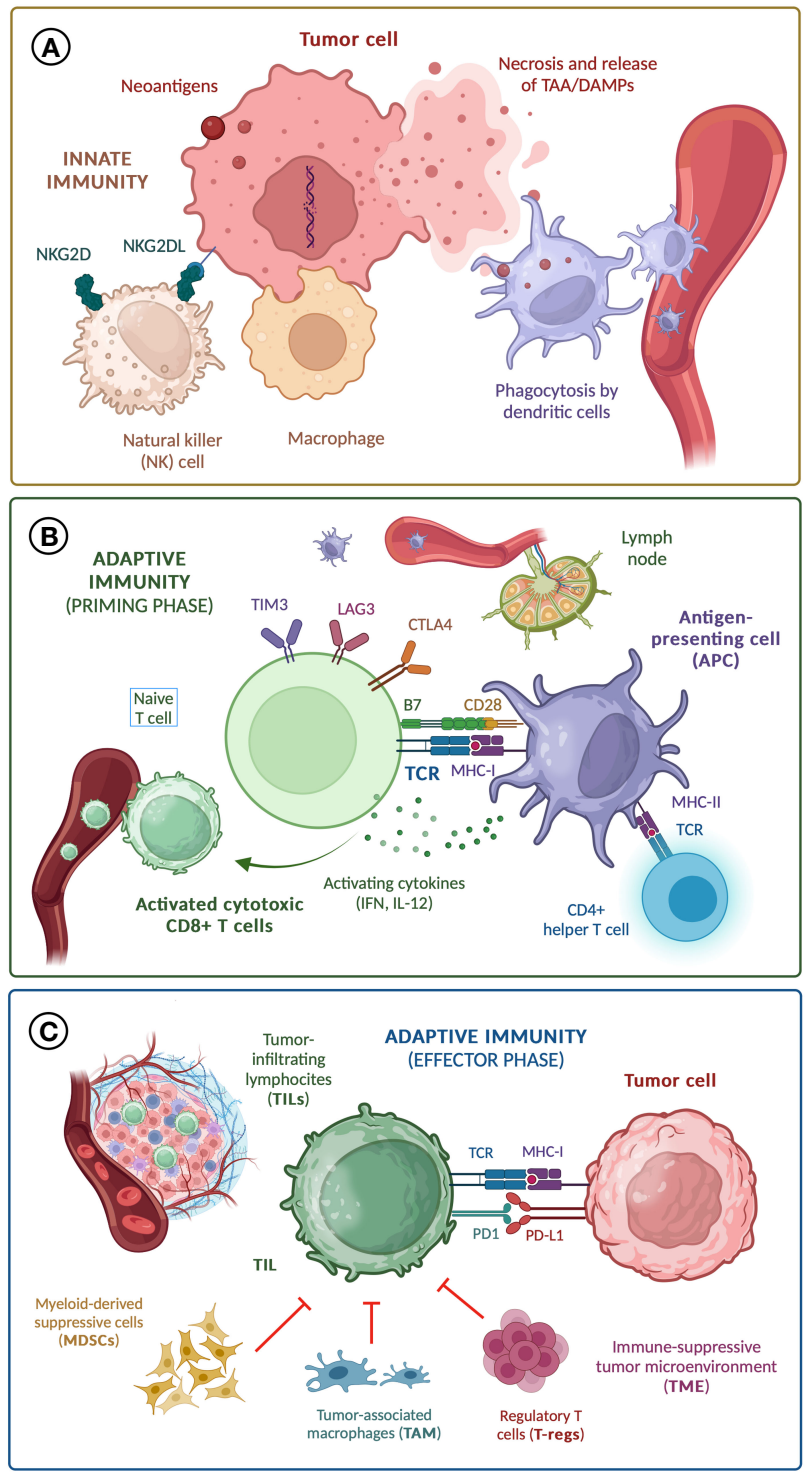
Mechanisms of anti-tumor response; **(A)** Innate immunity; **(B)** Adaptive immunity (priming phase); **(C)** Adaptive immunity (effector phase). TAA, tumor-associated antigen; DAMPs, damage-associated molecular patterns; TCR, T cell receptor; MHC, major histocompatibility complex; IL-12, interleukin-12; IFN, interferons.

The antitumor response is controlled by negative feedback mechanisms performed by molecules known as ‘immune checkpoints’, both in the priming phase -including CTLA-4, LAG-3 and TIM-3- and in the effector phase -mainly programmed cell death protein 1 (PD-1), activated by ligands (PD-L1) expressed both by cells from the tumor and TME- (10). The mechanisms of the antitumor response and potential immune biomarkers are shown in **Figure 1**.

The ability to avoid the immune system is a hallmark of malignant cells (11). Some of the most relevant mechanisms of immune evasion in solid tumors are the upregulation of immune checkpoints (12) -which sets the rationale for the use of immune checkpoint inhibitors (ICI)-, the loss of MHC-I or other molecules with a key role in antigen presentation (13), the production of cytokines (IL-6, IL-10, TGF-β) that lead to an immunosuppressive TME (14), and the activation of oncogenic routes that promote T-regs infiltration -such as the indoleamine 2,3-dioxygenase (IDO) pathway (15)- or inhibit CD8+ T-cell trafficking to the tumor site -such as the Wnt/β-catenin pathway (16)-. Recent data suggest that remote transference of biomolecular cargoes from malignant to healthy cells, mediated by exosomes, may also play a crucial role in misleading the mechanisms of antigenic recognition, thus contributing to immune tumor evasion (17).

Since the FDA approval of anti-CTLA4 and anti-PD1 therapy for advanced melanoma in 2011 and 2015, respectively, ICI alone or in combination with other therapies have transformed the therapeutic landscape of nearly all solid tumors (18). However, there are several other emerging strategies to enhance the immune antitumor response, some of them with promising results in ICI-refractory tumors, which will surely increase the relevance of immunotherapy in cancer treatment throughout the following years.

### 1.2 Basis of adoptive cell therapy

Adoptive cell therapy (ACT) encompasses several techniques that use the T lymphocytes themselves, after a process of artificial modification or genetic engineering, to improve their antitumor activity (19). This implies the extraction of autologous T lymphocytes from the patient, and their manipulation and amplification *in vitro*. Meanwhile, the patient undergoes treatment with lymphodepleting chemotherapy (CT) -usually fludarabine plus cyclophosphamide- to annihilate ineffective and immune-suppressing lymphocytes. After this process, the improved cell product is reinfused into the patient.

The difference between different ACT modalities lies in the characteristics of T cell modification *in vitro*: CAR-T and TCR gene-modified T cells are genetically engineered to incorporate modified membrane receptors with a high affinity for selected tumor antigens. However, in contrast to hematologic malignancies, solid tumors are composed of polyclonal cell populations with huge antigenic heterogeneity, which hampers the efficacy of antigen-targeted therapies. TIL therapy is based on the activation and expansion of infiltrating T cells extracted from the tumor itself, which are intrinsically reactive against tumor antigens, setting an interesting rationale for its use against the changing and heterogeneous cell population of solid cancers.

## 2 CAR-T cells, a role in solid cancers?

### 2.1 Introduction

CAR-T cells are genetically modified lymphocytes that incorporate a chimeric antigen receptor (CAR) composed by three parts: an extracellular domain with a single-chain fragment variable (scFv) that allows antigen recognition, a transmembrane domain -linked to the extracellular part through a *spacer*-, and an intracellular domain. This includes a CD3 complex -which activates the *downstream* signaling pathways- and several costimulatory domains (usually CD28 and/or 4-1BB) that intensify the cytoplasmatic activity of T cells unchained by antigenic recognition (20). In recent years, innovations in the structure and manufacturing of CAR-T cells have led to significant improvements in their clinical efficacy, especially with the development of fourth-generation CAR-T cells (21). Fifth generation CARs equipped with three costimulatory domains and able to secrete anti-PDL1 scFv blockade molecules, targeted against B cell maturation antigen (BCMA) have shown heightened antitumor efficacy and decrease of T cell exhaustion in patients with multiple myeloma (22).

Nanobodies or single domain antibodies (VHH) have recently been exploited as an alternative to scFvs for antigen-targeting domains on T cell surface, based on numerous advantages including their small size, high affinity, specificity and stability (23). VHH-based CD19-redirected CAR-T cells have shown similar expansion rate, cytotoxicity and anti-tumor reactions when compared with their scFv-based counterparts (24).

Due to their molecular structure, CAR-T cells only recognize extracellular antigens, and are particularly efficient when their scFv has a high affinity for the targeted protein -and it is homogeneously expressed by tumor cells-. This explains the efficacy of CAR-T cells in patients with leukemia and lymphoma, which comprise a clonal population of cells that uniformly express certain antigens -such as CD19- on their membrane (25). Since 2018, tisagenlecleucel and axicabtagene-citoleucel have EMA approval for the treatment of B-cell LLA and refractory non-Hodgkin lymphoma, and in 2020, brexucabtagene-autoleucel was approved by the EMA for mantle lymphoma, achieving complete response rates of over 50% in heavily pre-treated patients (26).

The outstanding results of CAR-T cells in hematologic malignancies have led to their investigation in solid tumors, mainly using overexpressed epithelial antigens as targets. The epithelial growth factor receptor (EGFR), HER2, carcinoembryonic antigen (CEA), mesothelin and soluble antigen GD2 have been frequent targets of CAR-T therapies, although many other antigens have been the object of preclinical studies (27).

### 2.2 Clinical outcomes

Nearly 500 clinical trials evaluating CAR-T cells in solid tumors have been registered, most of them in Asian population, and many still ongoing (28). Most completed studies are phase I/II trials that have reported modest results, with only occasional and generally brief clinical responses. Clinical research on CAR-T cells has mainly focused on glioblastoma (GBM), sarcoma, neuroblastoma, and gastrointestinal cancer.

In 2016, Brown et al. (29) reported the case of a GBM patient with an 8-month complete response (CR) after IL13-targeted CAR-T therapy, although further research has failed to confirm these results (30). Her2 may be another interesting target for CAR-T cells in GBM; in a clinical trial with 17 patients, 1 partial response (PR) (lasting 9 months) and 7 cases of stable disease (SD) (ranging from 2 to 29 months) were reported (31). EGFR-targeted CAR-T cells have been evaluated in GBM in two clinical trials, with negative results (32, 33).

Her2-targeted CAR-T cells have also been tested in sarcoma patients. In a phase I/II study including 19 patients with Her2+ recurrent or refractory sarcoma of several histological subtypes, 4 SD were observed [3 osteosarcoma and 1 small round cell desmoplastic tumor) (34). In neuroblastoma, at least three clinical trials have evaluated the efficacy of GD2-targeted CAR-T cells -based on the efficacy of anti-GD2 monoclonal antibodies such as dinutuximab- (35), with promising results (3 CR among 19 patients (36), 4 PR among 8 patients (37), and 5 SD among 11 patients (38)].

As for gastrointestinal (GI) cancer, several antigens have been evaluated as potential targets of CAR-T therapy. In a phase I trial including 23 patients with several GI tumors treated with CD133-targeted CAR-T cells, 3 PR (2 pancreatic and 1 hepatocellular carcinoma [HCC]) and 14 SD were observed (39). Zhan et al. (40) evaluated CAR-T therapy targeting Claudin 18.2 (CLDN 18.2) in 11 patients with CLDN 18.2-positive gastric or pancreatic carcinoma, reporting 1 CR, 3 PR and 5 SD. EGFR-CAR-T therapy has mainly been evaluated in biliopancreatic tumors, with promising results. Liu et al. (41) conducted a phase I study including 14 patients with refractory advanced pancreatic carcinoma, reporting 4 PR and 8 SD; in a phase I study by Guo et al. (42) with 19 patients (14 cholangiocarcinoma, 5 gallbladder carcinoma), 1 CR and 10 SD were observed. Glypican-3 (GPC3)-targeted (43) and CEA-targeted (44) CAR-T cells have shown modest activity in HCC and colorectal cancer, respectively.

Mesothelin-targeted intrapleural CAR-T cells have been evaluated -in combination with ICI- in 14 patients with malignant mesothelioma and non-small cell lung cancer (NSCLC), with promising results (2 CR, 5 PR, and 4 SD) (45). In NSCLC, a phase I study showed clinical activity of EGFR-CAR-T cells, with 2 PR and 5 SD among 11 patients (46), and ROR1-directed CAR-T cells showed preliminary positive results in ROR1+ tumors (4 PR among 6 patients) (47). CAR-T therapy has obtained modest results for metastatic castration-resistant prostate cancer (mCRPC) -targeting prostate specific membrane antigen (PSMA)- (48). Evidence for CAR-T therapy in other solid tumors is even scarcer and mainly comes from preclinical studies (49).

**Figure 2:**
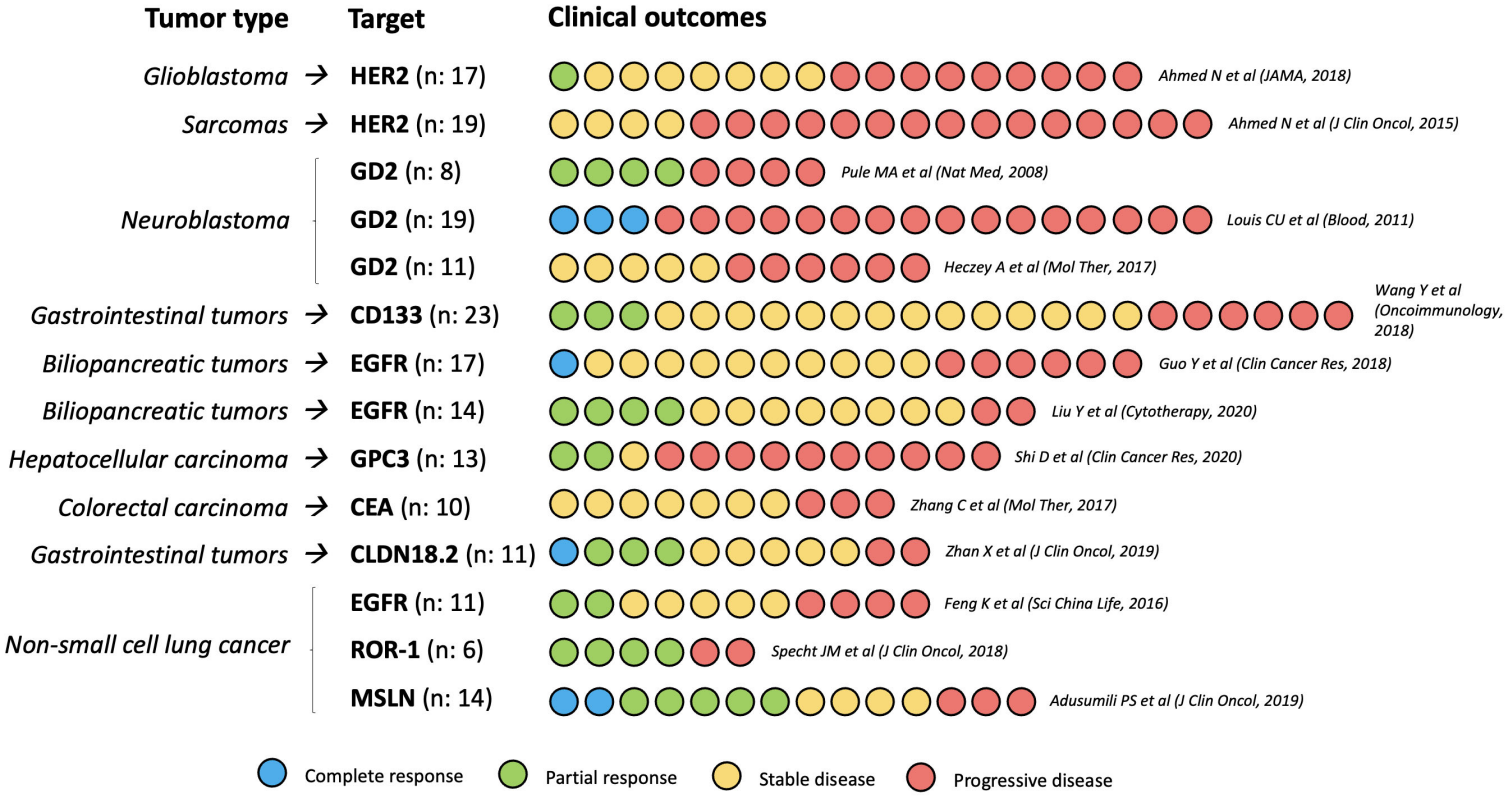
Clinical outcomes of the most relevant CAR-T cells phase I/II trials in solid tumors. N, number of participants in each study.

The outcomes of the most relevant clinical trials that evaluated CAR-T cells in solid tumors are summarized in **Figure 2**.

## 3 TCR-engineered T cells, more of the same?

### 3.1 Introduction

T cells expressing an engineered T-cell receptor (TCR-T-cells) represent an alternative modality of ACT, with several advantages compared to CAR T therapy (50). By using engineered TCR -instead of CAR-, T cells can recognize not only membrane proteins but also intracellular antigens presented by MHC molecules, covering a wider repertoire of tumor neoantigens and therapeutic targets. Intrinsic features of T cells, such as high antigen sensitivity and the use of physiological signaling pathways, improve TCR-T-cells antitumor functions and reduce the risk of *on-target off-tumor* (OTOT) toxicity.

However, TCR-T-cells also have some disadvantages compared to CAR-T cells, such as their weaker avidity for target antigens and their limitation to a certain human leukocyte antigen (HLA) haplotype, which restricts the number of patients that potentially benefit from each modified TCR. In addition, as antigen-targeted modified T lymphocytes, all the limitations of CAR-T therapy in solid tumors -antigenic heterogeneity, difficulty of T cell trafficking, and detrimental effects of immunosuppressive TME- are also applicable to TCR-T-cells (51).

### 3.2 Clinical outcomes

In 2006, Morgan et al. (52) published the results of the first trial with TCR-T-cells in solid tumors, targeting MART-1 in 17 patients with metastatic melanoma (with 2 PR). Since then, at least three other phase I/II clinical trials have evaluated MART-1-targeted TCR-T-cells in advanced melanoma -all using retrovirus as vectors-, and have reported variable results, with ORR ranging from 0% to 30% (53–55). In one of these trials (53), protein gp100 was also evaluated as a target in 16 patients, observing 1 sustained CR (>14 months) and 2 PR (ORR: 18.8%). TCR-T-cell-related toxicity was similar in all these studies, with a predominance of skin toxicity (23-94%) and a low incidence of CRS (<15%).

The New York esophageal squamous cell carcinoma (NY-ESO)-1 antigen is a promising target both in melanoma and in some sarcoma immunogenic subtypes -particularly synovial sarcoma, with NY-ESO-1 overexpression in 80% of patients (56)-. In a phase II trial with 38 melanoma and synovial sarcoma patients treated with NY-ESO-1 TCR-T-cells, there was an ORR of 57.9%, including 5 maintained CR and several long-term PR, with no severe toxicity related to TCR-T-cells (57). Similar results were obtained in two other phase I studies, with an incidence of CRS of 10% in one of them (58, 59). Two other phase I/II trials evaluated NY-ESO-1 TCR-T-cells in patients with synovial sarcoma -using lentivirus as a vector instead of retrovirus-, obtaining similar results (ORR 30% to 50%), with an incidence of CRS of 41.7% in one of them (60, 61). NY-ESO-1 was also used as TCR-T-cells target in a small phase I trial including 3 patients with different solid tumors, with negative results (62).

Other cancer-tesis antigens, such as MAGE family proteins, have been evaluated as TCR-T-cell targets in multi-tumor phase I/II trials. A phase I trial evaluated MAGE-A3-targeted TCR-T-cells in 17 patients with solid tumors and observed an ORR of 23.5% (including 1 sustained CR) (63). Morgan et al. (64) treated 9 solid tumors patients (including 7 synovial sarcomas) with MAGE-A3-targeted cells, obtaining an ORR of 56% (including 1 sustained CR), but with severe toxicity (ICANS in 3 patients and 2 treatment-related deaths). In a phase I trial with 38 patients treated with MAGE-A4-TCR-T-cells, 50% of CRS was observed (65), and another trial with MAGE-A3-TCR-T-cells was suspended after 2 treatment-related deaths (66). Cross-reactions against proteins normally expressed by nervous system cells -such as EPS8L2- have been hypothesized as the source of severe neurotoxicity and toxic deaths reported by MAGE-TCR-T-cells trials (67).

MAGE-targeted TCR-T-cells have obtained negative or modest results in NSCLC (68) and esophageal cancer (69). Similarly, significant toxicity has been observed in studies targeting other proteins that are not exclusively expressed by tumor cells. For example, a phase I trial evaluating CEA-targeted TCR-T-cells in patients with colorectal cancer was suspended due to severe colitis in 100% of the patients (70).

The treatment of virus-related tumors is an interesting approach for TCR-T-cells, since targeting specific viral antigens, which are expressed by infected tumor cells but not by normal tissues, should avoid the problem of cross-reactions and OTOT toxicity. In fact, two trials have evaluated TCR-T-cells targeted against human papillomavirus (HPV) carcinogenesis-related proteins (E6 and E7) in HPV+ tumors, without any relevant toxicity and interesting clinical outcomes (ORR of 16.7% and 50%, respectively) (71, 72). Meng et al. evaluated TCR-T-cells against hepatitis B virus (HBV) in 8 patients with HBV+ HCC, observing 1 prolonged PR and only one case of liver toxicity (73). Veatch et al. (74) tested TCR-T-cells against Merkel carcinoma polyomavirus (MCPyV) in 5 patients with refractory Merkel cell carcinoma, reporting 1 PR and no significant adverse events. However, given that virus-related tumors represent a small proportion of advanced solid cancers, other strategies are needed to overcome TCR-T-cells cross-reactions and OTOT toxicity.

In relation to this, an exciting hypothesis is the use of neoantigens -byproduct of tumor somatic mutations, not present in non-malignant tissues- as tumor-restricted and immunogenic targets of TCR-T-cells. In fact, some studies suggest that long-term responses to immune checkpoint inhibitors are mediated by neoantigen-reactive effector T cells (75), providing a rationale for combining TCR-T-cells and ICI. Unfortunately, a phase I trial exploring the effectiveness of personalized neoantigen-targeted TCR-T-cells in 16 patients with advanced solid tumors has obtained disappointing results (51).

Occasional responses to TCR-T-cells have been observed in other tumors. Leidner et al. (76) reported a partial response with KRAS G12D-targeted TCR-T-cells (lasting >6 months) in a patient with advanced pancreatic adenocarcinoma. Kim et al. (77) reported a partial response to TP53-targeted TCR-T-cells in a patient with metastatic breast cancer.

The outcomes of published TCR-T-cells clinical trials for solid tumors have been summarized in **Table 1**.

**Table 1:** Clinical trials with published results of TCR-T-cells in solid tumors.

Clinical trial	Target	Vector	N	Age range	Clinical responses	AEs related to TCR-T-cells
Melanoma						
**Morgan et al. (phase I) (** 52)	MART-1	Retrovirus	17	20-58	2 PR (20-21 m); ORR: 11.8%	None
** Johnson et al. (phase II) (** 53)	MART-1	Retrovirus	20	24-60	6 PR (3-17 m); ORR: 30%	Skin rash (70%), uveitis (55%), hearing loss (50%)
	Gp100	Retrovirus	16	25-62	1 CR (>14 m), 2 PR (3-4 m); ORR: 18.8%	Skin rash (94%), hearing loss (31%), uveitis (25%)
** Chodon et al. (phase II) (** 54)	MART-1	Retrovirus	13	40-61	7 SD (3-6 m); ORR: 0%	Skin rash (23%), CRS (15%)
** Rohaan et al. (phase I/II) (** 55)	MART-1	Retrovirus	12	43-74	2 PR (4-7 m); ORR: 16.7%	Skin rash (83%), hearing loss (33%), uveitis (17%), CRS (8%)
Melanoma and sarcoma						
** Robbins et al. (phase I) (** 57)	NY-ESO1	Retrovirus	17	19-61	2 CR (>20 m), 7 PR (3-18 m); ORR: 52.9%	None
** Robbins et al. (phase II) (** 58)	NY-ESO1	Retrovirus	38	19-65	5 CR (24 to >58 m), 17 PR (3 to >47 m); ORR: 57.9%	None
** Nowicki et al. (phase I) (** 59)	NY-ESO1	Retrovirus	10	24-66	2 PR (9-51 m); ORR: 20%	CRS (10%)
Synovial sarcoma						
** D’Angelo et al. (phase I/II) (** 60)	NY-ESO1	Lentivirus	12	18-51	1 CR (8 m), 5 PR (4-18 m); ORR: 50%	CRS (41.7%)
** Ramachandran et al. (phase I/II) (** 61)	NY-ESO1	Lentivirus	30	NE	9 PR (2-13 m); ORR: 30%	NE
Gastrointestinal cancer						
** Parkhurst et al. (phase I) (** 70)	CEA	Retrovirus	3	43-55	1 PR (6 m); ORR: 33%	Severe colitis (100%)
** Kageyama et al. (phase I) (** 69)	MAGE-A4	Retrovirus	10	43-73	ORR: 0%	None
** Leidner et al. (phase I) (** 76)	KRAS G12D	Retrovirus	1	71	1 PR (> 6 m); ORR: 100%	None
HPV+ tumors (cervical cancer, HNSCC)						
** Doran et al. (phase I/II) (** 71)	HPV16-E6	Retrovirus	12	32-70	2 PR (3-6 m); ORR: 16.7%	None
** Nagarsheth et al. (phase I) (** 72)	HPV16-E7	Retrovirus	12	31-65	6 PR (3-9 m); ORR: 50%	None
NSCLC						
** Blumenschein et al. (phase I) (** 68)	MAGE-A10	Lentivirus	11	46-72	1 PR (6 m); ORR: 9%	ICANS (9.1%)
HBV-related hepatocellular carcinoma (HCC)						
** Meng et al. (phase I) (** 73)	HBV	Electroporation	8	46-67	1 PR (27 m); ORR: 12.5%	Liver toxicity (12.5%)
Merkel cell carcinoma						
** Veatch et al. (phase I) (** 74)	MCPyV	Lentivirus	5	NE	1 PR (NE); ORR: 20%	None
Metastatic breast cancer						
** Kim et al. (phase I) (** 77)	TP53	Retrovirus	1	NE	1 PR (6 m); ORR: 100%	CRS
Multi-tumor						
** Morgan et al. (phase I/II) (** 64)	MAGE-A3	Retrovirus	9	21-71	1 CR (>15 m), 4 PR (4 to >12m); ORR: 56%	ICANS (33%) (2 deaths)
** Linette et al. (phase I) (** 66)	MAGE-A3	Lentivirus	2	57-63	ORR: 0%	Toxic death (100%)
** Lu et al. (phase I) (** 63)	MAGE-A3	Retrovirus	17	25-66	1 CR (>29 M), 3 PR (4 to >18 m); ORR: 23.5%	Hepatitis (12%)
** Hong et al. (phase I) (** 65)	MAGE-A4	Lentivirus	38	31-78	9 PR (NE); ORR: 23.7%	CRS (50%)
** Stadtmauer et al. (phase I) (** 62)	NY-ESO1	Lentivirus	3	62-66	ORR: 0%	None
** Foy et al. (phase I) (** 51)	Neoantigens	Electroporation	16	36-70	ORR: 0%	CRS (6%), ICANS (6%)

AEs, adverse events; CR, complete responses; PR, partial responses; ORR, objective response rate; NE, not specified; m, months; CRS, cytokine release syndrome; ICANS, immune effector cell-associated neurotoxicity syndrome; HPV, human papillomavirus; HNSCC, head and neck squamous cell carcinoma; HBV, hepatitis B virus; MCPyV, Merkel carcinoma polyomavirus.

## 4 Challenges and future strategies

### 4.1 Challenges and future strategies

Both CAR-T and TCR-T cells harbor inherent limitations for the treatment of patients with solid tumors, which may explain the significantly worse clinical outcomes than those obtained with CAR-T therapy in hematologic malignancies.

#### 4.1.1 Antigenic heterogeneity

In contrast to the clonal nature of lymphomas and leukemias, solid tumors -particularly in the context of metastatic disease- are characterized by the progressive acquisition of somatic mutations that lead to polyclonal expansion of different cellular lineages, giving rise to genomic instability and antigenic heterogeneity.

Significant intratumoral heterogeneity in neo-epitope expression and clonal expansion of the adaptive immune system in distant regions of the same disease have also been demonstrated in hepatitis B virus (HBV)-related liver cancer (78). In Her2+ breast cancer, spatial transcriptomic studies have shown intra-patient heterogeneity in the expression of gene signatures that determine cellular interactions with T lymphocytes and other immune cells (79). O’Rourke et al. (33) studied the expression of several antigens in tumor cells from 7 GBM patients, before and after a single infusion of EGFR-targeted CAR-T cells, observing in all of them a significant decrease in EGFR expression, but an important increase in several other antigens with known immunosuppressor functions (CD8, GRZMB, CD25, IDO1, PDL1, and FoxP3). These results shed light on how antigen-specific T cell therapies might quickly promote the selection of resistant cellular subclones with immunosuppressive activity.

T cells with multi-antigenic recognition have been proposed as a potential strategy to overcome this problem. Some preclinical studies have suggested that CAR-T cells with bispecific adapters can facilitate the eradication of antigenically different tumors (80). Moving a step forward, CAR-T cells equipped with synthetic Notch (synNotch) receptors might be able to induce CAR expression only after the recognition of tumor-specific antigens, creating precise prime-and-kill recognition circuits (81). Although these are promising strategies, they have not yet been evaluated in clinical trials, and further research is required to assess their feasibility.

#### 4.1.2 On-target off-tumor toxicity

Most antigens targeted by CAR-T or TCR-T cells in solid tumors are not specific to cancer cells and are also expressed on non-malignant tissues, leading to potential OTOT toxicity. In addition to the usual adverse events observed in patients with hematologic malignancies, such as cytokine release syndrome (CRS) and immune effector cell-associated neurotoxicity syndrome (ICANS), which are generally manageable (82), the OTOT effects of these cell therapies add significant toxicity and are usually dose-limiting in patients with solid tumors.

The release of perforin and granzymes following T cell activation is assumed to play a key role in OTOT cytotoxicity, although the upregulation of T cell-surface pro-apoptotic molecules (such as FAS ligands) might also contribute to tissue destruction (83). Acute respiratory distress, digestive hemorrhage, and severe mucocutaneous toxicity have been reported as OTOT effects in several clinical trials, particularly with Her2 (84)-, CLDN18.2 (85)- and EGFR (41, 42)-targeted CAR-T cells. Interestingly, no OTOT toxicity has been reported for anti-GD2 CAR-T cells in patients with diffuse midline gliomas (86) despite the fact that GD2 is expressed in healthy brain tissue (87). Although little is known about the threshold for antigen recognition (88), this suggests that CAR-T therapy may be feasible without significant OTOT effects in cases with different levels of antigen expression between tumor and healthy cells.

Some additional theoretical strategies to overcome OTOT toxicity include the modulation of scFv affinity and/or CAR architecture, the locoregional administration of CAR-T cells to avoid systemic effects, the development of engineering approaches to exogenously control CAR-T cell activity, and the design of protein-based logic-circuit strategies to restrict CAR-T cell activation (89). Further research is needed to assess the clinical feasibility of these approaches and discover predictive biomarkers of severe toxicity.

#### 4.1.3 T cells trafficking

Ensuring contact between CAR-T cells and tumor cells is not a problem in leukemias and lymphomas, since malignant cells concentrate within the blood and lymph nodes; however, it is a significant obstacle in solid metastatic tumors, particularly those with infiltration of immune-privileged organs -such as the central nervous system-. Several studies have shown that metastatic lesions from solid cancers have significantly lower lymphocytic infiltration than primary tumors, suggesting that the loss of T cell trafficking to the tumor site is a relevant mechanism for immune escape and may facilitate tumor progression (90).

In patients with metastatic melanoma, Harlin et al. (91) showed that the expression of certain cytokines (CCL2, CCL3, CCL4, CCL5, CXCL9, and CXCL10) is significantly higher in lesions enriched with TILs than in those with poor lymphocytic infiltration, suggesting that these molecules play an important role in T cell recruitment. Targeting the tumor vasculature and microenvironment to modulate the chemotactic response is an exciting research topic to improve CAR-T cell trafficking to solid tumors (92).

#### 4.1.4 Immunosuppressive TME

In addition to hampering T cell trafficking, the TME has inhibitory effects on the lymphocytes that get to infiltrate the tumor site, which is an obstacle for CAR T cell function. In addition, sustained exposure to tumor antigens and inflammatory signals is thought to progressively mitigate the function and proliferation of modified T cells, leading to CAR T cell ‘exhaustion’. Targeting T cell intrinsic pathways (PD-1/PD-L1 axis, TOX/NR4A, TGF-β, CBL-B), using CRISPR technology to modulate the surface expression of CAR, and uncoupling antigen recognition from CAR activation signaling, are exciting approaches under research to overcome exhaustion and improve CAR T cells clinical outcomes in solid tumors (93).

## 5 TIL therapy, the hope for ACT in solid tumors?

### 5.1 Introduction

*Ex vivo* expanded tumor-infiltrating lymphocytes (TILs) from different solid cancers share a composition of oligoclonal effector T cells that are reactive against a heterogeneous repertoire of tumor-associated antigens (94). This establishes the rationale for TILs artificial expansion and activation *in vitro* (out of the TME detrimental influence) and their subsequent reinfusion -together with stimulating cytokine IL-2- into a more favorable environment, after chemical depletion of immunosuppressive cells.

Not being a specific antigen-targeted therapy and using naturally ‘selected’ tumor-reactive lymphocytes, TIL therapy may theoretically overcome the problems of antigenic heterogeneity, tumor trafficking, and *on target off tumor* toxicity that limit the effectiveness of CAR-T and TCR-T therapies against advanced solid tumors.

### 5.2 Clinical outcomes in melanoma

TIL therapy has mainly been evaluated in advanced melanoma. Since the first positive studies conducted by Rosenberg (95–97), several phase I/II trials have obtained clinical responses with expanded TILs -alone or in combination with total body irradiation- with a significant variability in the IL2 dosage, the number of infused cells, and the intensity of lymphodepletion (98–101). All these studies were performed before the large-scale expansion of ICI and targeted therapy as the standard of care for advanced melanoma.

In ASCO 2020, Sarnaik et al. (102) communicated the results of a phase II trial evaluating cryopreserved autologous TIL therapy lifileucel (LN-144) in patients with metastatic melanoma in progression to anti-PD1 +/- anti-CTLA4 therapy (and BRAF/MEK inhibitors in *BRAF*-mutant tumors). Among the 66 evaluable patients, there were 2 CR and 22 PR (ORR 36.4%), with the median duration of response not reached at 18.7 months. A reduction in tumor burden was observed in 81% of patients. Responses were demonstrated regardless of the *BRAF* mutational status, PD-L1 expression, and tumor location. Objective responses were observed in patients with brain and liver metastases, baseline bulky disease, elevated lactate dehydrogenase (LDH) levels, and prior anti-PD1 treatment. The safety profile was consistent with the known toxicities of the lymphodepletion and IL-2 regimens.

Two years later, Rohaan et al. (103) published the first phase III trial of ACT for solid tumors, comparing TIL therapy with ipilimumab in patients with stage IIIC/IV melanoma. A total of 168 patients were randomly assigned in a 1:1 ratio to receive TILs (at least 5 x 10^9^ cells, preceded by lymphodepleting CT, followed by IL2 at high-doses of 600.000 IU/kg) or ipilimumab (84 patients in each group). 89% of the patients had received previous systemic therapy, most of them adjuvant or first-line anti-PD1 antibodies. Median progression-free survival (mPFS) was significantly higher in the TIL group (7.2 vs 3.1 months; hazard ratio [HR] 0.50; *p <*0.001), as well as the ORR (49% vs 21%). The median overall survival was 25.8 months (vs 18.9 months in the ipilimumab group).

All the above-mentioned studies have been conducted on patients with cutaneous melanoma. A phase II trial evaluated TILs in 21 patients with metastatic uveal melanoma, with 1 sustained CR and 6 PR (ORR of 35%) (104). Interestingly, 3 of these responders (43%) had previously received immunotherapy with anti-PD1 and/or anti-CTLA4 agents, without any clinical benefit. These results demonstrate that TIL therapy merits further research in non-cutaneous melanoma, especially considering its refractory nature to ICI and other systemic treatments.

The outcomes of the published clinical trials of TIL therapy have been summarized in **Table 2**.

**Table 2:** Clinical trials with published results of TIL therapy in solid tumors.

Clinical trial	Treatment	IL-2 dose	N	Age range	Clinical responses	AEs related to TIL therapy
Melanoma						
**Rosenberg et al. (phase I) (** 95)	TILs (*no LD)	High	20	21-59	1 CR (>13 m), 10 PR (2-9 m); ORR: 55%	Nausea (55%), CRS (50%), ICANS (30%), respiratory distress (10%)
**Rosenberg et al. (phase I) (** 96)	TILs (*partial LD)	High	86	11-70	5 CR (>20 m), 24 PR (mDR: 4 m); ORR: 34%	Nausea (43%), CRS (28%), ICANS (21%), respiratory distress (8%), toxic death (1.2%)
**Rosenberg et al. (phase II) (** 97)	TILs +/- TBI	High	93	16-75	20 CR (37 to >82 m), 32 PR (NE); ORR: 56%	1 toxic death (1.1%) (other data NE)
**Dudley et al. (phase I) (** 98)	TILs +/- TBI	High	35	11-70	3 CR (>7 to >14 m), 15 PR (2 to >30 m); ORR: 51%	Vitiligo* (37%), uveitis* (14%), respiratory distress* (9%), ICANS* (3%) (*only G3/G4)
**Ellebaek et al. (phase I/II) (** 99)	TILs	Low	6	36-62	2 CR (>10 and >30 m), 0 PR; ORR: 33%	Fatigue (100%), nausea (83%), diarrhea (83%), dermatitis (50%), allergic reaction (50%)
**Andersen et al. (phase I/II) (** 100)	TILs	Dec	24	25-68	3 CR (>22 to >47 m), 7 PR (>17 to >45 m); ORR: 42%	ICANS (8.3%), vitiligo (8.3%), respiratory distress (4.2%), renal failure (4.2%), diarrhea (4.2%), uveitis (4.2%), vasculitis (4.2%), 1 toxic death (4.2%)
**Goff et al. (phase II) (** 101)	TILs +/- TBI	High	101	18-65	24 CR (NE), 30 PR (NE); ORR: 54%	CRS (6.1%), cardiac arrhythmia (5.1%), RRT (3%), intubation (2%), 1 toxic death (1%)
**Sarnaik et al. (phase II) (** 102)	TILs	High	66	20-79	2 CR, 22 PR; ORR: 36.4%; mDR not reached at 18.7 m	Pyrexia* (16.7%), hypotension* (10.6%), CRS* (6.1%), fatigue* (1.5%) (*only G3-G4 events)
**Rohaan et al. (phase III) (** 103)	TILs (vs ipi)	High	84	26-74	17 CR, 24 PR; ORR: 49% vs 21%; mPFS: 7.2 m vs 3.1 m	100% G3-G4 AEs (vs 57%); CRS (84%), fatigue (68%), hypotension (41%), CLS (30%), vitiligo (11%), uveitis (8%), hearing loss (4%)
**Chandran et al. (phase II) *(uveal melanoma) (*** 104)	TILs	High	21	32-63	1 CR (>21 m), 6 PR (4-9 m); ORR: 35%	Dyspnea* (10%), cardiac arrhythmia* (5%), renal failure* (5%), thrombosis* (5%) (*only G3-G4 events). 1 toxic death (infection)
NSCLC						
**Creelan et al. (phase I/II) (** 105)	TILs + nivo (after PD on nivo)	Dec	13	38-75	1 CR (>18 m), 2 PR (>12 to >23 m) (*plus 11 SD with tumor reduction); ORR: 23%	Nausea (86%), skin rash (55%), diarrhea (55%), CRS (45%); total severe toxicity: 12.5%
**Schoenfeld et al. (phase II) (** 106)	TILs	NE	24	40-74	1 CR (>21 m), 5 PR (4 of them >8 m); ORR: 25%	NE
Cervical cancer						
**Jazaeri et al. (phase II) (** 107)	TILs	NE	27	NE	1 CR (NE), 9 PR (*plus 2 unconfirmed PR); ORR: 44%	NE
HNSCC						
**Jimeno et al. (phase II) (** 108)	TILs + pembro	NE	9	NE	1 CR, 3 PR; ORR: 44%; mDR not reached at 6.9 m	NE
HPV+ epithelial tumors (HNSCC, cervical and anal cancer)						
**Stevanovic et al. (phase II) (** 109)	TILs	High	29	30-63	2 CR (>53 and >67 m), 5 PR (3-5 m); ORR: 24%	Metabolic disorders (41.4%), nausea (20.7%), hypoxia (27.6%), dyspnea (13.8%), ICANS (3.4%)
Breast cancer						
**Zacharakis et al. (phase II) (** 110)	Pembro > TILs > pembro	High	6	35-67	1 CR (>66 m), 2 PR (6-10 m); ORR: 50%	NE
Multi-tumor						
**Kverneland et al. (phase I/II) (** 111)	Ipi > TILs > nivo	Low	25	39-66	2 PR (3-7 m); ORR: 8%	Fever* (16%), PS drop* (12%), dyspnea* (8%), transaminase elevation* (4%) (*only G3-G4)
**O’Malley et al. (phase II) (** 112)	TILs + pembro	NE	31	24-73	Melanoma (n: 8): 3 CR, 4 PR; ORR: 87.5% HNSCC (n: 13): 1 CR, 5 PR; ORR: 46.2% Cervical (n: 10): 1 CR, 4 PR; ORR: 50%	NE

AEs, adverse events; CR, complete responses; PR, partial responses; ORR, objective response rate; mDR, median duration of response; NE, not specified; m, months; CRS, cytokine release syndrome; ICANS, immune effector cell-associated neurotoxicity syndrome; LD, lymphodepletion; TBI, total body irradiation; dec, decrescendo; RRT, renal replacement therapy; ipi, ipilimumab; CLS, capillary leak syndrome.

### 5.3 Clinical outcomes in other solid tumors

Although TIL therapy has not yet achieved robust results in non-melanoma tumors, some phase I/II clinical trials have shown promising data in other immunogenic malignancies, such as NSCLC, cervical cancer, and HNSCC.

In a phase I/II trial, Creelan et al. (105) evaluated the efficacy of autologous TILs in combination with anti-PD1 nivolumab in 20 patients with advanced NSCLC following progression to nivolumab monotherapy. Among the 13 evaluable patients, there were 3 objective responses (2 CR -both ongoing >18 months-, 1 PR, and 8 SD with tumor reduction). Interestingly, one of the patients with sustained CR was *EGFR*-mutant (exon 19 deletion), which is a known predictor of anti-PD1 failure (113), suggesting that NSCLC subtypes that are commonly refractory to ICI might not be resistant to TIL therapy. Further research is required to assess whether TIL therapy could have a re-sensitizing effect for ICI use.

A phase II trial evaluated TIL monotherapy (LN-145) in 28 patients with advanced pretreated NSCLC, observing 1 CR (>21 months) and 5 PR (4 of them >8 months) among 24 evaluable patients (ORR 25%) (106).

TIL therapy also has promising results in a phase II trial with patients with advanced cervical cancer, observing 1 CR and 11 PR (2 of them unconfirmed by study criteria) (ORR: 44%) (107). Another study evaluated TILs in 29 patients with HPV+ epithelial tumors (HNSCC, cervical and anal cancer), with an ORR of 24% (including 2 CR, ongoing after 53 and 67 months) (109).

O’Malley et al. (112) conducted a multi-tumor trial with TIL therapy plus pembrolizumab in 31 patients (13 HNSCC, 10 cervical cancers, and 8 melanoma), with positive results (ORR 46.2%, 50%, and 87.5%, respectively). This TIL+ICI combination has also been evaluated in small phase II trials in HNSCC (108) and metastatic breast cancer (110), with promising results (ORR 44% and 50%, respectively). Interestingly, the second study included a patient with hormone-positive breast cancer who achieved a sustained CR (>5 years). However, a limitation of these trials is that some patients, especially those with ICI-responding tumors such as melanoma and HNSCC, might have responded to anti-PD1 blockade itself, making the role of TIL therapy difficult to assess. The sequential use of ipilimumab, TIL therapy, and nivolumab in different solid tumors was evaluated in a phase I/II trial of 25 patients, with modest results (2 PR ranging from 3 to 7 months) (111).

### 5.4 Challenges for clinical practice

Despite the promising results of TIL therapy in clinical trials, many practical and economic challenges have limited its large-scale implementation (114).

TIL manufacturing begins with a surgical resection of tumor tissue, preferably from metastases accessible with minimally invasive surgery. To date, the generation of TILs has been equally successful regardless of the resected lesion site (115). Enzymatic digestion and/or mechanical fragmentation of the surgical sample is followed by culture of the fragments in IL-2 containing media, a process that might take 2-6 weeks. This minimally expanded or “young” TILs are massively expanded using high doses of IL-2, anti-CD3, and irradiated peripheral blood mononuclear cells (PBMCs) or ‘feeder cells’, within a 2-weeks rapid expansion protocol (REP). The expanded TILs are eventually reinfused into the lymphodepleted patients, followed by high-dose bolus IL2 (114).

This is an expensive and logistically complex process that requires highly specialized facilities, protocolized procedures, and qualified technical staff. These are important limitations for the widespread application of TIL therapy, particularly in developing countries. Grade 3-4 adverse events, which appear in virtually all patients treated with TIL therapy, mainly following lymphodepletion and IL2 administration, also require highly specialized management. The significant treatment toxicity, together with the long duration of the manufacturing process, are substantial obstacles to the successful application of TIL therapy in patients with refractory metastatic tumors that often lead to rapid clinical deterioration.

To date, TIL therapy has been evaluated in young, fit patients with an ECOG performance status (PS) of 0-1. Even in this selected population from clinical trials, it is often found that only a small proportion of the screened patients can finally receive treatment. For example, in a phase II trial of TIL therapy in advanced breast cancer, only 6 of the 46 screened patients (13%) received TILs infusion (110). No residual or evaluable disease after resection, inadequate material for screening, negative or weak isolation of lymphocytes in the resected samples, clinical progression, PS deterioration, and lymphodepletion-related severe toxicity were common causes of treatment failure.

Lastly, not only the economic expenses but also the individualized nature of TIL therapy, which differs from conventional commercial drugs, are important challenges for TIL therapy regulatory approval. Previous experience with CAR-T cells in hematologic malignancies will surely smooth the way for dealing with the health-economic aspects of TIL therapy and its implementation in clinical practice.

## 6 Conclusion

The clinical outcomes of adoptive cell therapy (ACT) in solid tumors are conditioned by biological aspects that substantially differ from those of hematologic malignancies, such as their antigenic heterogeneity, immunosuppressive microenvironment, and immune scape ability. Dozens of phase I/II trials with CAR-T cells have only led to sporadic, usually short-term clinical responses, although novel strategies, such as multiantigenic recognition, SynNotch receptors, and CAR modulation through CRISPR technology, are promising approaches to improve their efficacy. Despite the inherent limitations of antigen-targeted treatments, TCR-engineered T-cells have several advantages over CAR-T cells and have shown promising results in certain tumors, such as melanoma and synovial sarcoma with NY-ESO1 overexpression. In the opinion of the authors, the treatment of HPV+ and other virus-related malignancies, as well as the use of neoantigens as targets, are exciting fields of TCR-T-cells research.

Conceptually different from other ACT modalities, TIL therapy is based on the extraction, *ex vivo* expansion, and stimulation of naturally reactive tumor-infiltrating lymphocytes, followed by their reinfusion into lymphodepleted patients. TILs have shown positive results in advanced melanoma, with a recent positive phase III trial that has proved their superiority to ipilimumab in anti-PD1 refractory disease. The efficacy of TIL therapy, alone or in combination with checkpoint inhibitors, has also been demonstrated in other solid tumors including NSCLC, cervical cancer, and HNSCC. Strategies to selectively expand neoantigen-reactive TILs or genetically modify the expanding cells through CRISPR technology are exciting lines of research, to improve the efficacy of TIL therapy in patients with melanoma and extend its clinical benefit to other malignancies. However, numerous clinical and practical limitations that currently hinder its large-scale implementation still need to be overcome.

## Author contributions

VA: Writing – original draft, Writing – review & editing. MS: Writing – review & editing. JP: Writing – review & editing. JC: Writing – review & editing. DR: Writing – review & editing. PG: Writing – review & editing. JCC: Writing – review & editing. CG: Writing – review & editing. CGQ: Writing – review & editing. PP: Writing – review & editing. JM: Writing – review & editing. AC: Writing – review & editing. AS: Writing – review & editing.
